# The effect of ethanol and acetaldehyde on brain ependymal and respiratory ciliary beat frequency

**DOI:** 10.1186/2046-2530-2-5

**Published:** 2013-03-25

**Authors:** Claire Mary Smith, Priya Radhakrishnan, Kulvinder Sikand, Chris O’Callaghan

**Affiliations:** 1 Department of Respiratory Medicine, Portex Unit, Institute of Child Health, University College London and Great Ormond Street Hospital, 30 Guilford Street, London, WC1N 1EH, UK; 2 Department of Infection, Immunity and Inflammation, Division of Child Health, University of Leicester, Leicester, LE2 7LX, UK

**Keywords:** Ependyma, Cilia, Alcohol, Ciliary beat frequency, Acetaldehyde

## Abstract

**Background:**

Ethanol has been shown to stimulate the beat frequency of respiratory cilia at concentrations encountered during social drinking, while one of its metabolites, acetaldehyde, has been shown to cause a marked decrease in ciliary beat frequency. The aim of this study was to determine whether short-term exposure to ethanol stimulated ependymal cilia and whether exposure to acetaldehyde had a toxic effect on ependymal and respiratory cilia.

**Methods:**

Using *ex vivo* rat ependymal brain slice and human nasal brush biopsy models, we investigated the effect of exposure of cilia to various concentrations of ethanol and acetaldehyde at either 37°C or 24°C. Ciliary beat frequency was measured using digital high-speed video analysis.

**Results:**

Exposure of ependymal and respiratory cilia to control, 0.1%, 0.5% and 1% ethanol solutions resulted in a maximal increase of 15% in the ciliary beat frequency from baseline values, compared with the control of 6%. A one-way analysis of variance comparing the mean slopes for the three concentrations of ethanol and control showed no significant differences between the groups (*P* >0.05). Exposure of ependymal and respiratory cilia to 100 and 250 μM acetaldehyde solutions resulted in a maximal increase of 15% in the ciliary beat frequency from baseline, compared with the control of 12%. A one-way analysis of variance performed to compare the mean slopes in these groups showed no significant differences (*P* >0.05).

**Conclusions:**

Short-term exposure of brain ependymal and respiratory cilia to the concentrations of ethanol likely to be encountered during episodes of heavy drinking and to acetaldehyde at concentrations well above those encountered by man did not have a significant effect on ciliary beat frequency.

## Background

Ethanol and its metabolite acetaldehyde have been shown to have significant effects on respiratory cilia. Although extremely high concentrations of ethanol have been linked to slowing of respiratory cilia, Maurer and Liebman found that concentrations of alcohol which may be achieved from social drinking had no detrimental effect on respiratory cilia [1]. Indeed, they reported stimulation of ciliary beating after exposure to low levels of ethanol. Sisson also found that ethanol rapidly stimulated the respiratory ciliary beat frequency at concentrations as low as 10 μM and found that suppression of ciliary beat frequency did not occur until levels exceeded 1,000 μM [2]. Acetaldehyde has been shown to be toxic to bovine respiratory cilia, markedly slowing the ciliary beat frequency or causing complete ciliary stasis [3]. It has been suggested that chronic ethanol exposure would be likely to result in cumulative dysfunction if the rate of adduct formation by acetaldehyde exceeded the ability of the cells to replace damaged proteins.

Motile cilia are also found in other parts of the body in man including the ependyma, a single layer of ciliated cells that line the cerebral ventricles and aqueducts and the central canal of the spinal cord [4]. Ependymal cells have approximately 40 motile cilia that beat approximately twice as fast as respiratory cilia [5] moving cerebrospinal fluid in the direction of cerebrospinal fluid flow.

We were keen to determine the effect of ethanol, and particularly acetaldehyde, on ciliated ependymal cells because other toxic insults that inhibit ciliary activity, such as intracerebroventricular antisense knockdown of Gα_i2_[6] and metavanadate, induce hydrocephalus in rats [7]. Functional abnormalities of ependymal cilia are also important during development as shown by humans and animals with ciliary defects who are at increased risk of hydrocephalus [7-15].

Another suggestion is that ciliated brain ependymal cells, unlike those from the respiratory tract, may not be replaced efficiently following damage [16].

The aim of our study was to determine the effect of ethanol and its metabolite acetaldehyde on the ependymal ciliary beat frequency.

## Methods

### Sample preparation

The brains of Wistar rats (9 to 15 days of age) were dissected following sacrifice. Brain slices from the floor of the fourth ventricle were prepared immediately after sacrifice and mounted in a well containing 4 ml of medium 199 with Earl’s salts (pH 7.4; plus penicillin 50 u/ml and streptomycin 50 μg/ml) and kept on ice until the study began. Human ciliated samples were obtained by brushing the inferior nasal turbinate with a 2-mm cytology brush (Keymed, Southend-on-Sea, UK) as previously described. Ethical approval for the collection of nasal epithelial cells was given by the Leicestershire Ethical Review Committee. Cells were dislodged from the brush by shaking it in 1 ml of medium 199 containing 100 IU/ml penicillin and streptomycin (Calbiochem, Nottingham, UK) and were kept on ice until the study began. For the experiment, the well was placed in a purpose-built environmental chamber that was thermostatically controlled to keep the fluid surrounding the ciliated sample at 37°C or 24°C. The chamber was humidified to 75 to 80% to prevent evaporation from the well during the 4-hour study period. No reduction in the volume of ethanol or acetaldehyde was seen during the experiment. Ciliary movement was observed using an inverted Nikon microscope and a ×50 or ×100 lens [17,18].

### Measurement of ciliary beat frequency

Beating ciliated strips were recorded by a high-speed video camera (Kodak EktaPro Motion Analyser, Model 1012, San Diego, CA, USA) or a Motion Pro X4 digital high-speed video camera (Lake Image Systems, USA) at a rate of 400 frames per second. The camera allowed video sequences to be downloaded at reduced frame rates, allowing the ciliary beat frequency to be determined directly by timing a given number of individual ciliary beat cycles. At each time point of the study, the ciliary beat frequency was measured at four different areas along each ciliated strip. Only intact, undisrupted, ciliated strips in excess of 100 μm were studied. Using this system our beat frequency measurements are very reproducible, with the components of variance for intra-subject and inter-subject variability being only 1% and 3.8%, respectively, of the total variation.

### Exposure to ethanol

Ciliated ependymal or nasal epithelial strips were incubated, in medium 199, for 30 minutes in the environmental chamber at a temperature of 37°C or 24°C. Baseline readings of ciliary beat frequency were made and the surrounding cell culture fluid exchanged for one of the study concentrations of ethanol in medium 199, or medium 199 alone, preheated to 37°C or 24°C. The ciliary beat frequency was measured after 15, 30 and 60 minutes (for ependymal cilia) and hourly for up to 4 hours for respiratory cilia. The concentrations of ethanol studied were 0.1% (*n* = 5), 0.5% (*n* = 8) and 1% (*n* = 6) for ependymal cilia and 0.5% (*n* = 4) and 1% (*n* = 4) for respiratory cilia. These concentrations range in molarity from 17 to 172 mM ethanol. Matched controls in medium 199 were studied for each concentration of ethanol (controls, *n* = 21).

### Exposure to acetaldehyde

The study protocol was similar to that used for exposure to ethanol. Baseline measurements of ciliary beat frequency were made following incubation in medium 199 for 30 minutes at 37°C or 24°C. Medium 199 surrounding the cells was exchanged for a known concentration of acetaldehyde (100 or 250 μM) in medium 199 or a control solution of medium 199, preheated to 37°C or 24°C. Sisson and colleagues have shown marked ciliary slowing following exposure to similar concentrations of acetaldehyde, within minutes [3]. Pilot studies were conducted, measuring the ciliary beat frequency every few minutes. When no change in ependymal ciliary beat frequency occurred, the study design was changed to one where the ciliary beat frequency was measured after 30, 60, 90, 120, 150 and 180 minutes. The concentrations of acetaldehyde studied were 100 μM (*n* = 6) and 250 μM (*n* = 6) using medium 199 alone as a control solution (*n* = 4).

This study was repeated using respiratory cilia exposed to 100 μM (*n* = 4) and 250 μM (*n* = 4) concentrations of acetaldehyde using medium 199 alone as a control solution (*n* = 4). Baseline measurements of ciliary beat frequency were made following incubation in medium 199 for 30 minutes at 37°C or 24°C. Ciliary beat frequency was measured and hourly for up to 4 hours.

#### Statistical analysis

Statistical analysis was performed using GraphPad Prism 5 (GraphPad, San Diego, CA, USA). Data were analysed using one-way analysis of variance with Dunnet post-test comparison with the control (untreated group) for each temperature and tissue tested. Data were expressed as means ± standard deviation. *P* <0.05 was taken as the threshold for statistical significance.

## Results

### Ependymal cilia

**Figure 1:**
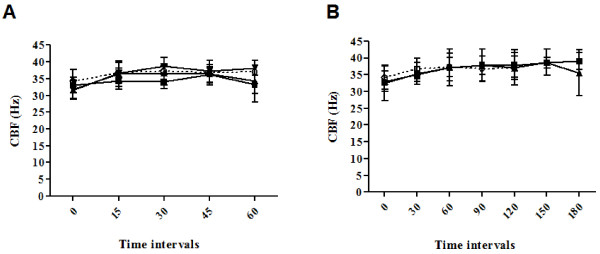
**Effect of ethanol and acetylaldehyde on rat brain ependymal ciliary beat frequency at 37°C.** (**A**) Effect of ethanol on rat brain ependymal ciliary beat frequency (CBF). Three different concentrations of ethanol (0.1%, filled squares; 0.5%, filled triangles; 1%, open squares) and a control (open circles) were studied. (**B**) Effect of acteylaldehyde on rat brain ependymal CBF. Two different concentrations of acetylaldehyde (100 μM, filled squares; 250 μM, filled triangles) and a control (open circles) were studied. Data represent the mean and standard deviation of 5 to 13 repeat experiments.

The ciliary beat frequency from individual brain slices following exposure to ethanol or control solutions are shown in Figure 1A.

There were three different concentrations of ethanol (0.1%, 0.5% and 1%) and a control studied. The one-way analysis of variance comparing the mean slopes for the three concentrations of ethanol and control studied showed no significant differences between the four groups (*P* = 0.21). The linear trend tested for in the four group means was not significant (*P* = 0.10).

Exposure of ependymal cilia to control, 0.1%, 0.5% and 1% ethanol solutions resulted in a +6.1%, +6.8%, +11% and +15% increase, respectively, in the ciliary beat frequency from baseline values. To test whether analysing the percentage change from baseline made a difference to the interpretation of our results, the slope of the regression line of percentage change against time was calculated for each slice. The one-way analysis of variance comparing the mean slopes in the four groups showed no significant differences (*P* = 0.1). The linear trend tested for in the four group means was also not significant (*P* = 0.5). The conclusions were the same as in the previous analysis.

The ciliary beat frequency from individual brain slices following exposure to acetaldehyde or control solutions are shown in Figure 1B.

For the two concentrations of acetaldehyde (100 and 250 μM) and the control group studied, the slope of the regression line of frequency against time was calculated for each slice. A one-way analysis of variance performed to compare the mean slopes in these three groups showed no significant differences (*P* = 0.9). The linear trend tested for in the three group means was not significant (*P* = 0.8).

Exposure of ependymal cilia to control, 100 μM and 250 μM acetaldehyde solutions resulted in a +12%, +12.2% and +15% increase, respectively, in the ciliary beat frequency from baseline. The analysis was again repeated after expressing the data as the percentage change from the baseline value. The one-way analysis of variance comparing the mean slopes in the three groups showed no significant differences (*P* = 0.7). The linear trend tested for in the three group means was not significant (*P* = 0.7).

### Respiratory cilia

**Figure 2:**
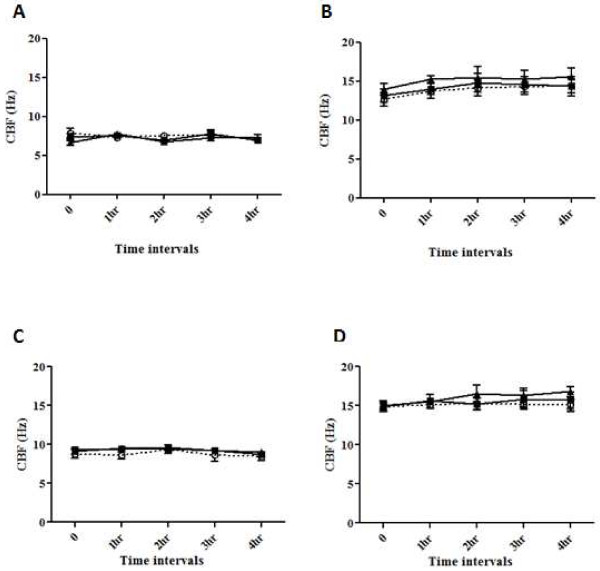
**Effect of ethanol and acetylaldehyde on human respiratory epithelial ciliary beat frequency.** (**A**), (**B**) Effect of ethanol on human respiratory epithelial ciliary beat frequency (CBF). Three different concentrations of ethanol (0.1%, filled squares; 0.5%, filled triangles; 1%, open squares) and a control (open circles) were studied at (**A**) 24°C and (**B**) 37°C. Data represent the mean and standard deviation of five different experiments. (**C**), (**D**) Effect of acetylaldehyde on human respiratory epithelial CBF. Two different concentrations of acetylaldehyde (100 μM, filled squares; 250 μM, filled triangles) and a control (open circles) were studied were studied at (**C**) 24°C and (**D**) 37°C. Data represent the mean and standard deviation of five different experiments.

The ciliary beat frequency from strips of intact human nasal epithelial cells following exposure to ethanol or control solutions is shown in Figure 2A,B.

There were two different concentrations of ethanol (0.5% and 1%) and a control studied. The one-way analysis of variance comparing the mean slopes for the two concentrations of ethanol and control studied showed no significant differences between the three groups at 24°C (*P* = 0.39) and 37°C (*P* = 0.15).

Exposure of nasal epithelial cilia to control, 0.5% and 1% ethanol solutions resulted in a −11.3%, –4.9%, and +8.0% change, respectively, in the ciliary beat frequency from baseline values at 24°C. At 37°C, exposure of nasal epithelial cilia to control, 0.5% and 1% ethanol solutions resulted in a 14.0%, 9.3%, and 12.0% increase, respectively, in the ciliary beat frequency from baseline values. The one-way analysis of variance comparing the mean slopes in the three groups showed no significant differences at 24°C (*P* = 0.39) or 37°C (*P* = 0.15).

The ciliary beat frequency nasal epithelial cilia following exposure to acetaldehyde or control solutions are shown in Figure 2C,D.

For the two concentrations of acetaldehyde (100 and 250 μM) and the control group studied, the slope of the regression line of frequency against time was calculated for each slice. A one-way analysis of variance performed to compare the mean slopes in these three groups showed no significant differences (*P* = 0.9). The linear trend tested for in the three group means was not significant (*P* = 0.8).

Exposure of nasal epithelial cilia to control, 100 μM and 250 μM acetaldehyde solutions resulted in a −3.5%, –6.1% and −1.8% decrease, respectively, in the ciliary beat frequency from baseline at 24°C. At 37°C, exposure of nasal epithelial cilia to control, 100 μM and 250 μM acetaldehyde solutions resulted in +1.8%, +5.5%, and +11.4% increases, respectively, in the ciliary beat frequency from baseline values. The analysis was again repeated after expressing the data as the percentage change from the baseline value. The one-way analysis of variance comparing the mean slopes in the three groups showed no significant differences (*P* = 0.32). The linear trend tested for in the three group means was not significant (*P* = 0.7).

## Discussion

Neither ethanol nor acetaldehyde, at concentrations ranging from those found in the blood during social drinking to concentrations considered lethal, had a significant effect on ependymal or nasal ciliary beat frequency. The lack of response of ependymal and nasal cilia to short-term exposure to acetaldehyde is of interest as this is different to the response of bovine respiratory cilia, where a marked effect on ciliary beat frequency was seen at similar concentrations [3]. The study on bovine respiratory cilia found that an acetaldehyde concentration of 60 μM reduced the ciliary beat frequency from 14 to 5 Hz within 30 seconds and that a concentration of 250 μM caused complete ciliary stasis within 2 minutes of exposure. In contrast, we found a nonsignificant rise in ependymal ciliary beat frequency over the concentrations of acetaldehyde studied. Although our results of short-term exposure to acetaldehyde are reassuring, they cannot predict long-term effects.

Over 90% of the ingested dose of ethanol is oxidised by different metabolic pathways, the most important being the alcohol dehydrogenase route. Alcohol dehydrogenase is a single dependent enzyme that oxidises ethanol to acetaldehyde. Acetaldehyde is produced in biologically significant quantities following the metabolism of ethanol, with levels as high as 50 μM being reported in the venous blood of alcoholics [19]. After its formation, acetaldehyde is oxidised to acetate by aldehyde dehydrogenase. Apart from being oxidised to acetate, acetaldehyde may bind covalently to proteins *in vivo*, resulting in acetaldehyde adducts. Aldehydes are highly reactive molecules and are recognised as important toxins in biological systems. Acetaldehyde may bind covalently to numerous proteins, resulting in protein and cellular dysfunction [20].

Sisson and colleagues hypothesised that acetaldehyde impaired bronchial epithelial ciliary motion by inhibiting ciliary dynein ATPase activity through the formation of acetaldehyde adducts with ciliary proteins [3]. Acetaldehyde binding was demonstrated to occur with dynein heavy chains and with tubulin, and closely paralleled ATPase inhibition. The study concluded that acetaldehyde inhibits ciliary dynein ATPase activity, and also binds to ciliary proteins, including dynein and tubulin, which are critical for motion. The marked difference in response to acetaldehyde between bovine respiratory, human respiratory and rat ependymal cilia is of interest given the similarity in structure and function of cilia from various sites and animals.

Maurer and Liebman studied the effects of concentrations of alcohol that may be achieved from social drinking on the ciliary beat frequency [1]. The ciliary beat frequency was stimulated at ethanol concentrations ranging from 0.01% up to but not including 0.1%, unchanged at 0.5 and 1%, and slowed at 2%. In keeping with this, Sisson found that ethanol caused a rapid stimulation of bovine respiratory cilia with concentrations as low as 10 μM ethanol and that no detectable decreases in ciliary beat frequency were found until ethanol concentrations exceeded 1,000 μM [2]. A stereospecific nitric oxide synthetase inhibitor, *N*-monomethyl-l-arginine completely blocked ethanol-induced stimulation of ciliary beat frequency. The frequency could be restored by adding either sodium nitroprusside, which is a direct nitric oxide donor, or l-arginine. These results indicate that ethanol, at clinically relevant concentrations, stimulated the release of nitric oxide by airway epithelium, resulting in an upregulation of ciliary beat frequency. Further studies showed this required the dual action of both cyclic AMP and cyclic guanosine monophosphate-dependent protein kinases, PKA and PKG, in ciliated cells [21,22]. Wyatt and colleagues also found a stimulation of respiratory cilia when rats were fed for 1 week with 36% alcohol [23].

Although we saw a trend towards an increase in ependymal ciliary beat frequency on exposure to increasing concentrations of ethanol and acetaldehyde, these increases in frequency did not reach statistical significance. The reason for the lack of significant stimulation to ethanol and the lack of toxicity on exposure to acetaldehyde is unclear, although both the tissue and animals used in our experiments were different. The ependymal cilia we studied were part of fresh long strips of ependyma attached to neuronal tissue. In this preparation, access of ethanol and acetaldehyde to the ciliated cells observed would have been via the apical surface of the cells. In the study on bovine respiratory cilia [3], cell-rich aggregates were prepared overnight and were studied at 24°C, a temperature at which cilia are capable of increasing their beat frequency considerably. Our experiments were conducted at much higher temperature (37°C). We chose this temperature because ependymal cilia are not exposed to lower temperatures *in vivo* and our interest was in the potential clinical effect. Perhaps the ependymal cilia in our studies were beating at a near-maximum rate, making any response to stimulation less obvious. This suggestion is supported by the lack of effect we have found on exposure of ependymal cells to the β_2_-agonists isoprenaline and forskolin at 37°C (unpublished observations COC).

Although our results for short-term exposure to acetaldehyde are reassuring, they may not predict the effect of long-term exposure. Further investigation of such exposure is required because damaged ciliated ependymal cells are not thought to be replaced and because functional abnormalities of ependymal cilia have been linked to the development of hydrocephalus [6,7].

Whether the tissues studied could actively metabolise acetaldehyde is unclear. People with inadequate ALDH2 activity are not able to effectively metabolise acetaldehyde and those affected develop significant side effects following ingestion of a relatively small amount of alcohol. We could find no literature on the ability of rat ependymal cells or bovine respiratory epithelial cells to metabolise acetaldehyde. Whether variations in the ability of ciliated epithelial cells, from different species or from different sites in the body, to metabolise acetaldehyde result in different levels of toxicity merits investigation.

## Conclusions

In summary, short-term exposure of rat brain ependymal cilia and human respiratory cilia to blood concentrations of ethanol at levels likely to be encountered during episodes of heavy drinking and of acetaldehyde at concentrations well above those encountered by man did not have a significant effect on ependymal ciliary function. These results are in contrast to studies on bovine bronchial respiratory tissue, where ciliary stimulation was seen following exposure to ethanol and marked depression of ciliary activity was seen following exposure to acetaldehyde.

## Competing interests

The authors declare that they have no competing interests.

## Authors’ contributions

CMS analysed the results and edited the manuscript. PR measured ciliary function and conducted the experiments. KS measured ciliary function and conducted the experiments. CO’C designed the study, analysed the results and wrote the manuscript. All authors read and approved the final manuscript.
